# Estimating deaths attributable to airborne particles: sensitivity of the results to different exposure assessment approaches

**DOI:** 10.1186/s12940-017-0213-9

**Published:** 2017-02-22

**Authors:** Simone Giannini, Michela Baccini, Giorgia Randi, Giovanni Bonafè, Paolo Lauriola, Andrea Ranzi

**Affiliations:** 1 Environmental Health Reference Centre, Regional Agency for Environmental Protection and Energy of Emilia-Romagna, Via Begarelli 13, 41121 Modena, Italy; 2 Department of Statistics, University of Bologna, Bologna, Italy; 3 Department of Statistics, Computer Science, Applications, University of Florence, Florence, Italy; 4 Department of Clinical Science and Community Health, University of Milan, Milan, Italy; 5 Institute for Health and Consumer Protection, Joint Research Centre of European Commission, Ispra, VA Italy; 6 Hydro Meteorological Service, Regional Agency for Environmental Protection and Energy of Emilia-Romagna, Bologna, Italy; 7 Regional Center for Environmental Modelling, Regional Agency for Environmental Protection of Friuli Venezia Giuli, Palmanova, Italy

**Keywords:** PM_10_, PM_2.5_, Health impact assessment, Natural mortality, Short-term effects

## Abstract

**Background:**

Epidemiological evidences support the existence of an effect of airborne particulate on population health. However, few studies evaluated the robustness of the results to different exposure assessment approaches. In this paper, we estimated short term effects and impacts of high levels of particulate matter with aerodynamic diameter ≤10 μm (PM_10_) and ≤2.5 μm (PM_2.5_) in the Emilia-Romagna region (Northern Italy), one of the most polluted areas in Europe, in the period 2006–2010, and checked if the results changed when different exposure definitions were used.

**Methods:**

Short-term impact of particles on population mortality was assessed, both considering the 9 provincial capitals of the Emilia-Romagna and the region as a whole. We estimated the effects of PM_10_ and PM_2.5_ on natural mortality by combining city-specific results in a Bayesian random-effects meta-analysis, and we used these estimates to calculate impacts in terms of attributable deaths. For PM_10_, we considered different definitions of exposure, based on the use of the air pollutant levels measured by different monitoring stations (background or traffic monitors) or predicted by a dispersion model.

**Results:**

Annual average concentrations of PM_10_ and PM_2.5_ exceeding the WHO limits of 20 and 10 μg/m^3^ were respectively responsible for 5.9 and 3.0 deaths per 100 000 inhabitants per year in the provincial capitals, during the period 2006–2010. The total impact in the region in 2010 amounted to 4.4 and 2.8 deaths per 100 000 for PM_10_ and PM_2.5_, respectively. The impact estimates for PM_10_ did not substantially change when the exposure levels were derived from background or traffic monitoring stations, or arose from the dispersion model, in particular when the counterfactual value of 20 μg/m^3^ was considered. The effect estimates appeared more sensitive to the exposure definition.

**Conclusions:**

A reduction in particle concentrations could have produced significant health benefits in the region. This general conclusion did not change when different exposure definitions were used, provided that the same exposure assessment approach was used for both effect and impact estimations. Caution is therefore recommended when using effect estimates from the literature to assess health impacts of air pollution in actual contexts.

## Background

Scientific evidence that ambient air pollution affects human health has increased since 2000, with several studies performed in the fields of epidemiology, clinical medicine and toxicology [1–4]. Epidemiological research has systematically documented the association of high air pollutant concentrations with the occurrence of a wide spectrum of both acute and chronic adverse effects on the health of population living in urban areas [5–7]. Large meta-analyses conducted in the United States and Europe indicate that exposure to air pollution levels currently observed in urban contexts is associated with short-term increase in mortality and morbidity. Evidence of an effect is substantial for particulate matter, in particular for particles ≤10 μm and ≤2.5 μm in diameter (PM_10_ and PM_2.5,_ respectively) [6, 8–17].

**Fig. 1:**
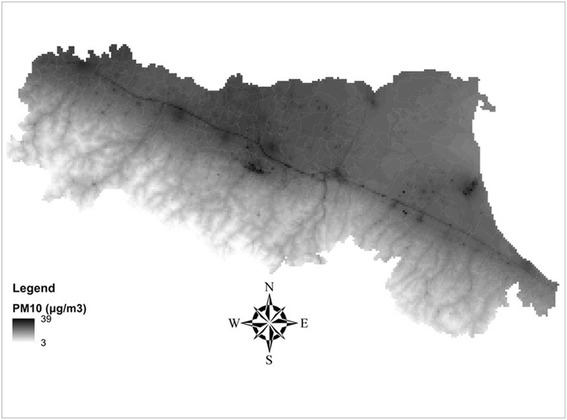
Annual mean levels of PM_10_ (μg/m^3^) in the Emilia-Romagna region, 2010

Different epidemiological studies conducted in various places around the world have led to consistent findings, supporting the causal interpretation of the effect measures and the absence of major bias [18]. This consistency provides the basis for reliable health impact assessments [19–22], which are crucial for addressing public health policies in a context where the magnitude of the effects is small (e.g. mortality from all causes was estimated to increase by 0.2–0.8% per 10 μg/m^3^ increase in the daily concentration of airborne particles [6, 8–17]) but exposure is widespread.

Several projects have considered short-term impact of air pollution in Italy during the last decades [8, 10, 11, 15, 23, 24]. The MISA study focused on 11 Italian cities for the period 1996–2002, reporting that around 1.5% of deaths from natural causes were attributable to PM_10_ levels greater than 11.9 μg/m^3^ [8]. The EpiAir2 project, studying data from 25 major Italian cities, highlighted that 0.9% and 0.8% of natural deaths were attributable to levels of PM_10_ and PM_2.5_ exceeding respectively the limits of 20 and 10 μg/m^3^ recommended by the World Health Organization (WHO) [24]. Another analysis on the air pollution effects in the Lombardy region for the period 2003–2006 found that 1.4% of natural deaths were attributable to annual average levels of PM_10_ higher than 20 μg/m^3^ [23].

This paper aims at contributing to the discussion on the short-term impact of air pollution on human health, providing an evaluation of the number of deaths attributable to high levels of PM_10_ and PM_2.5_ in the Emilia-Romagna region, one of the most polluted areas in Europe [25, 26]. The recent PM_10_ and PM_2.5_ concentration excesses observed in December 2015 and January 2016 in this region stressed the need of performing this kind of impact assessment, in order to inform policies of emissions reduction and plans for population’s health protection.

The paper also focuses on exposure assessment. Exposure assessment is preliminary to health impact assessment and using different models or approaches to quantify population exposure can produce very different results. In most studies, the impact of air pollution on health is evaluated by considering the air pollution levels measured by stations belonging to urban monitoring networks [19–22, 24]. This approach has the advantage of relying on observed concentrations, but it could lead to incorrect impact estimates if the monitor location does not allow detecting the actual population exposure. Moreover, it could be unfeasible if the interest is in extra-urban areas where data from monitors are usually unavailable. In this situation, models are needed to predict air pollutants concentrations [23, 27–29].

In this paper, we adopted and compared alternative exposure assessment approaches, considering air pollution levels measured by background or traffic monitoring stations or a combination of both, and using the air pollutant levels predicted for the entire region at a very fine spatial resolution using a model developed by the Environmental Protection Agency of the Emilia-Romagna region (ARPAE-ER) [30]. Our aim was not to draw conclusions about the best option to quantify population exposure, but to evaluate the sensitivity of the results, intended as the robustness of effect and impact estimates to different exposure assessment approaches.

## Methods

### Study population and data collection

The Emilia-Romagna region is located in the Po Valley, covers an area of 22 447 km^2^ in North-Eastern Italy and includes nine provinces and 348 municipalities, with 4.3 million inhabitants.

Daily counts of deaths from natural causes (ICD-9 001-799 or ICD-10 A00-R99) for the period 2006–2010 were collected for the population aged 35 years or over of the nine provincial capitals (Bologna, Modena, Parma, Reggio Emilia, Ravenna, Rimini, Ferrara, Forlì, and Piacenza), each one having a population of more than 100 000 inhabitants. Only deaths of individuals occurring in their town of residence were considered [15].

The total number of deaths in 2010 was also collected for all municipalities across the region. In this case, deaths occurring outside the city of residence were not excluded from the count. All mortality data were extracted from the Regional Health Information System and collected according to a common protocol.

For each municipality in the region, we considered the amount of total population and the percentage of the population of each census block from the available population census.

The daily time series of air pollution measurements, temperature and relative humidity for the period 2006–2010 for the nine provincial capitals were provided by the air quality and meteorological monitoring networks of the ARPAE-ER.

Estimates of the mean annual levels of PM_10_ and PM_2.5_ in the Emilia Romagna region for the year 2010 at 1 × 1 km spatial resolution were provided by ARPAE-ER using the NINFA-PESCO modelling suite (Fig. 1): a chemistry-transport model corrected through a Kriging approach, by using the observed data arising from the background monitoring stations located in the region [30].

### Exposure assessment

For each air pollutant, environmental data in the 9 provincial capitals were collected from monitors located in the city area. To satisfy measurement continuity over the study period, all monitors included in the study were required to have at least 75% complete information for each season (warm season: from April to September; cold season: from October to March). By averaging information from the selected monitors, we obtained a unique daily time series of exposure for each pollutant and city [31].

PM_10_ data for the period 2006–2010 were available for all cities except Ferrara (for Piacenza, the time series was limited to 2007–2010). For each city, three different definitions of exposure were considered for PM_10_: “background” exposure, based only on concentrations measured by background monitoring stations, “traffic” exposure, based only on concentrations measured by traffic monitoring stations, and “average” exposure, based on concentrations measured by both types of monitors used in the two previous calculations.

PM_2.5_ data for the period 2008–2010 were also collected; for Piacenza, Ferrara and Ravenna they were not available for the entire 3-year period. Only background monitoring stations were available for this pollutant.

To obtain homogeneous values of exposure for all the municipalities of the region, an annual average exposure in 2010 for both pollutants was calculated by integrating the air pollutant levels predicted in the 1 × 1 km cells by the NINFA-PESCO modelling suite. This model was calibrated using the air pollutant levels measured by the background monitors. Starting from the cell values, a population-weighted (PW) average was calculated for each municipality, assuming a uniform distribution of the inhabitants within each census block. The PW averages were obtained using ArcMap 10.1 software [32].

### Statistical analysis

The statistical analysis was performed in two steps. First, we estimated the regional short-term effects of the pollutants in the years 2006–2010, using data from the major cities in the region and assuming homogeneity of the effects during the study period; second, health impact assessment was carried out for the period 2006–2010 on the nine provincial capitals and for the year 2010 on all municipalities of the region.

#### Effect estimates

In order to estimate the effect of the two air pollutants on mortality, we firstly performed city-specific analyses on the nine provincial capitals according to the same protocol; then we combined the first stage city-specific estimates in a Bayesian random effects meta-analysis.

We estimated the short-term effects of PM_10_ and PM_2.5_ separately for each city, considering daily deaths of the resident population occurring inside the cities. This way, we avoided the possible bias of considering persons who did not experience the air pollutant exposure in the city.

We adopted the average of the current-day and of the previous-day concentrations (lag 0–1) as indicator of PM_10_ and PM_2.5_ exposure [10, 11, 23, 24]. We specified over-dispersed Poisson regression models on the daily counts of natural deaths, accounting for seasonality through an interaction term between year, month and day of week (this approach is equivalent to a time-stratified case-crossover approach) [33]. Analyses were also adjusted for temperature, population decrease during summer, holidays and influenza epidemic [15]. Regarding temperature adjustment, two different regression splines were fitted: one for temperatures below and one for temperatures above the city-specific median. The first spline was defined on the average temperature calculated over the previous 6 days (lag 1–6), whereas the second spline was defined on the lag 0–1 apparent temperature, which is a linear combination of temperature and relative humidity [15]. A three-level variable was included in the model to account for the population decrease during summer: this variable was equal to 2 in the 2-weeks centred on 15 August (Italian cities are largely deserted during these weeks), equal to 1 from 16 July to 31 August with the exception of the aforementioned 2-week period, and equal to 0 elsewhere. Dummy variables were introduced to model the effect of holidays and of influenza epidemics, defined according to the Italian national influenza surveillance system.

The effects of PM_10_ and PM_2.5_ were separately estimated from one-pollutant models. Analyses were performed using R 3.0.1 software [34].

As a second step, a Bayesian random-effects meta-analysis was specified to combine the city-specific estimates, and a sample from the joint posterior distribution of the model parameters was obtained using WinBugs software [35, 36]. Bayesian meta-analysis provided an estimate of the posterior overall effect, which is a combination of the first stage city-specific estimates, and an estimate of the *shrunken* city-specific effects, that borrow strength from all locations, while reflecting heterogeneity among cities [37]. The posterior distribution of the *I* ^*2*^ index, which represents the percentage of total variability explained by between-city heterogeneity, was also obtained.

The effect estimates were expressed as percentage variation in natural mortality associated with a 10 μg/m^3^ increase of exposure. The posterior distributions of the percentage increases were summarized in terms of posterior mean, 50 and 90% credibility intervals (the (1-α)% credibility interval (CrI) is defined as the interval between the (α/2)^th^ and the (1-α/2)^th^ percentiles of the posterior distribution) [38, 39].

For PM10, separated analyses were performed using the three measures of exposure based on different selections of the available monitoring stations (see Exposure assessment section).

For PM2.5 only the analysis on the “background” exposure (the only available one) was conducted.

#### Health impact assessment

Short-term impacts of high levels of PM_10_ and PM_2.5_ on mortality were estimated both at city and regional level. The impact was quantified in terms of attributable deaths (AD) and attributable community rate (ACR) (number of AD over the exposed population) per year [40, 41].

In order to estimate AD, the macro and micro approaches, proposed by Baccini et al., were used [23]. The macro approach allowed us to estimate the number of deaths attributable to annual levels of air pollutant (PM_10_ or PM_2.5_) exceeding a certain value *V*:

$$ A{D}_i={y}_i-{y}_{i0}={y}_i-{y}_i/ exp\left({\beta_i}^{*}\left({x}_i- V\right)\right), $$

where *i* labels the city, *y*_*i*_ is the observed annual number of deaths, *y*_*i0*_ is the baseline annual number of deaths at the counterfactual level *V*, *x*_*i*_ is the annual average level of air pollutant and *β*_i_^*^ is the coefficient expressing the effect of air pollution on a log scale. If *x*_*i*_ *< V*, *AD*_*i*_ was set to *0. AD*_*i*_ can be interpreted as the number of deaths which could have been prevented if the annual average of the air pollutant was equal to *V*.

In the micro approach, AD calculation was performed day-by-day, using the daily time series of mortality and air pollutant concentration: this analysis allowed us to evaluate the impact of exposure to daily peaks of air pollution. It should be noticed that, if the correlation between daily mortality and daily exposure is small, the macro approach approximates the results that one would obtain by applying a day-by-day micro approach after defining counterfactual daily values consistent with the counterfactual value defined for the annual average [23].

For the nine provincial capitals, we estimated the impact for the period 2006–2010 (2008–2010 for PM_2.5_) using the “background” exposure and the corresponding *shrunken* estimates of the air pollutant effect, when available. If for a city the *shrunken* estimate was not available, the overall meta-analytic estimate was employed [23]. For PM_10_ “average” and “traffic” exposures were also considered.

The impact at regional level was estimated for the year 2010. In this case, we considered the exposures predicted by the NINFA-PESCO model and AD calculation was carried out adopting the “background” overall meta-analytic estimate as effect estimate for all municipalities, except provincial capitals for which the *shrunken* estimates were used, if available. To estimate the impact at regional level, we considered all deaths, including those occurring outside the municipality of residence, in order to avoid impact in small municipalities without hospitals being underestimated.

##### Counterfactuals

We assessed the impact under different definitions of the value V, corresponding to different emission reduction scenarios. These scenarios were similar to those reported in a study conducted in the Lombardy region for the 2003–2006 period [23]. For PM_10_, the following reduction scenarios (RS) were defined:

- RS1-PM_10_: the annual average concentration does not exceed the WHO Air Quality Guideline value of 20 μg/m^3^ [42];
- RS2-PM_10_: the annual average concentration is equal to that observed at the “Febbio” monitoring station in the mountain town of Villa Minozzo (province of Reggio Emilia), a non-urban area;
- RS3-PM_10_: daily concentrations do not exceed the limit of 50 μg/m^3^ for more than 35 days per year, corresponding to the European Union (EU) limit for daily averages [43].

For PM_2.5_, the following RS was considered:

- RS1-PM_2.5_: the annual average concentration does not exceed 10 μg/m^3^ corresponding to the WHO Air Quality Guideline value [42].

While the impacts under the RS1 and RS2 scenarios were evaluated using the macro approach, the number of attributable deaths under the RS3-PM_10_ scenario was estimated by averaging 1 000 different pseudo-data obtained by constraining to 50 μg/m^3^ different random sets of days exceeding this value, so that the number of days with a concentration above the limit was set at 35 per year in each simulation. This calculation required the micro approach on daily data [23], so it was applied only for the nine provincial capitals, being daily data available only for these cities.

## Results

The total population of the 9 provincial capitals (Bologna, Modena, Parma, Reggio Emilia, Ravenna, Rimini, Ferrara, Forlì, and Piacenza) counted over 1.5 million; Bologna was the biggest city and Piacenza the smallest (Table 1). In these cities the air pollution levels measured by the traffic monitors were higher than those measured by the background monitors, Ravenna being the only exception. For this city, the greater level of air pollutant measured by the background monitor was possibly due to the fact that this monitor is located close to the harbor. Ravenna is a city near the sea, with a relevant industrial area and considerable transportation of inert materials due to the harbor activities. Considering the “background” exposure level during the period 2006–2010, all nine capitals exceeded the WHO annual limits of 20 μg/m^3^ for PM_10_ and 10 μg/m^3^ for PM_2.5_. Only Modena exceeded the EU limit of 40 μg/m^3^ for PM_10_ [43], when considering both the “traffic” and “average” exposure levels. For this reason, scenarios based on the EU limit of 40 μg/m^3^ for PM_10_ annual average level were not considered. Overall, in the nine capital cities the annual levels of PM_10_ obtained by averaging the cell values predicted by the NINFA-PESCO model were lower than those measured by the background monitors (29.8 μg/m^3^ vs 32.2 μg/m^3^). Differences were negligible for PM_2.5_. The PW average predicted annual concentrations of PM_10_ and PM_2.5_ over the region were 27.3 μg/m^3^ and 18.6 μg/m^3^, respectively.

**Table 1:** Population characteristics and air pollutant levels, Emilia Romagna region (2006–2010)

City	Population in 2011	Annual average of deaths (occurred in the municipality of residence)	Percentage of deaths occurred outside municipality of residence	PM_10_ concentration μg/m^3^				PM_2.5_ concentration μg/m^3^	
				Monitoring station 2006–2010 (mean ± sd)			PW-average level 2010 (mean)	Monitoring station 2006–2010 mean ± sd	PW-average level 2010 (mean)
				“background ” exposure	“average” exposure	“traffic” exposure		“background” exposure	
Piacenza	100,331	1,004	12.8	37.0 ± 21.8	38.5 ± 22.6	40.8 ± 24.4	33.1	24.9 ± 14.9^a^	22.5
Parma	175,895	1,709	8.6	34.8 ± 20.6	35.4 ± 20.2	36.4 ± 21.1	31.4	21.0 ± 15.8	20.6
Reggio Emilia	162,082	1,304	12.0	33.2 ± 19.6	37.1 ± 21.1	38.9 ± 22.2	31.0	22.6 ± 15.1	20.8
Modena	179,149	1,720	5.6	36.5 ± 23.1	41.0 ± 24.1	42.8 ± 24.7	32.7	21.7 ± 15.2	20.6
Bologna	371,337	3,845	15.0	25.8 ± 17.2	38.5 ± 21.6	38.5 ± 21.5	28.9	16.8 ± 12.3	15.3
Ferrara	132,545	1,577	6.0	27.2 ± 15.9 ^b^	37.6 ± 22.7	37.5 ± 22.7	27.9	20.8 ± 15.1^b^	20.5
Ravenna	153,740	1,328	10.5	30.8 ± 14.8	29.8 ± 15.5	28.9 ± 16.9	30.0	16.8 ± 11.5^c^	19.6
Forlì	116,434	940	20.2	29.4 ± 17.8	32.1 ± 18.3	35.0 ± 19.5	26.6	17.7 ± 13.2	19.5
Rimini	139,601	1,145	11.3	34.7 ± 19.8	34.9 ± 18.5	35.4 ± 18.3	27.4	21.9 ± 17.5	19.5
9 cities	1,531,094	14,572	11.6	32.2 ± 19.0	36.1 ± 20.5	37.1 ± 21.3	29.8	20.5 ± 14.5	19.2
Emilia Romagna region	4,342,135	44,844*		-			27.3	-	18.6

Monitoring period from: a) 14/09/2009; b) 20/11/2008 ; c) 03/04/2009

Abbreviations: *PW* population-weighted

*: this value includes deaths occurred outside the municipality of residence

When considering the “background” exposure, the pooled meta-analytic estimate of the percentage variation in natural mortality was 0.58 for each 10 μg/m^3^ increase in PM_10_ concentration (90% CrI: -0.09, 1.10) and 0.31 for each 10 μg/m^3^ increase in PM_2.5_ concentration (90% CrI: -0.96, 1.57) (Table 2). The heterogeneity among cities was low, but slightly higher for PM_10_ (*I* ^*2*^ = 2.77, 90% CrI: 0.08, 53.78) than for PM_2.5_ (*I* ^*2*^ = 1.30, 90% CrI: 0.03, 40.39). The shrunken percentage variations varied from 0.43 (Ravenna) to 0.71 (Piacenza) for PM_10_, and from 0.20 (Parma and Modena) to 0.47 (Reggio Emilia) for PM_2.5_.

**Table 2:** Shrunken city-specific effects^a^ and overall meta-analytic effect of PM_10_ and PM_2.5_ on natural mortality and corresponding 50 and 90% credibility intervals, Emilia-Romagna region (2006–2010)

City^b^	PM_10_			PM_2.5_^d^		
	% variation (Posterior mean)	50% Credibility Interval	90% Credibility Interval	% variation (Posterior mean)	50% Credibility Interval	90% Credibility Interval
Piacenza^c^	0.71	0.39, 1.08	−0.03, 2.23	-	-	-
Parma	0.53	0.23, 0.84	−0.32, 1.28	0.20	−0.32, 0.75	−1.23, 1.54
Reggio Emilia	0.64	0.32, 0.95	−0.15, 1.54	0.47	−0.09, 0.99	−0.92, 1.98
Modena	0.47	0.14, 0.77	−0.47, 1.15	0.20	−0.33, 0.76	−1.21, 1.56
Bologna	0.56	0.27, 0.85	−0.21, 1.25	0.35	−0.16, 0.86	−0.96, 1.66
Ravenna	0.43	0.05, 0.76	−0.22, 1.70	-	-	-
Forlì	0.62	0.31, 0.94	−0.22, 1.70	0.24	−0.33, 0.81	−1.26, 1.65
Rimini	0.64	0.34, 0.84	−0.12, 1.70	0.41	−0.17, 0.93	−0.99, 1.87
Overall - “Background” exposure	0.58	0.31, 0.84	−0.09, 1.10	0.31	−0.18, 0.79	−0.96, 1.57
I^2^ (%)	2.77	0.48, 14.61	0.08, 53.78	1.30	0.20, 7.57	0.03, 40.39
Overall - “Average” exposure	0.36	0.12, 0.60	−0.10, 0.82	-	-	-
I^2^ (%)	2.40	0.48, 11.96	0.09, 49.07			
Overall - “Traffic” exposure	0.36	0.12, 0.60	−0.23, 0.95	-	-	-
I^2^ (%)	2.24	0.46, 10.79	0.09, 46.60	-	-	-

^a^: Effects are expressed as percentage variations in natural mortality associated with an increase of 10 μg/m^3^ in PM_10_ or PM_2.5_ concentration at lag 0–1

^b^: Ferrara is not reported due to for this city the background levels of exposure were not available for the entire study period

^c^: Study period for PM_10_: 2007–2010

^d^: Study period: 2008–2010

In order to check the sensitivity of the effect estimates to the specific subset of cities included in the meta-analysis, we applied a leave-one-out approach. When excluding one at time each capital city from the meta-analysis, the overall percentage variation varied from 0.4 to 0.8% (data not reported) for PM_10_, that was in any case within the credibility interval of the overall meta-analytic effect.

The last three lines of Table 2 show the sensitivity analysis on different specifications of the exposure levels. The estimate of the overall meta-analytic effect based on “background” exposure was higher compared to the other two modalities. When PM_10_ exposure was defined using only traffic monitoring stations, the overall meta-analytic effect estimate was substantially unchanged compared to that obtained with the “average” exposure (Table 2).

Table 3 shows the short term impact of PM_10_ and PM_2.5_ on mortality in the provincial capitals during the period 2006–2010, evaluated under different emission reduction scenarios. The results refer to exposures assessed using background monitoring stations. Exceeding the WHO limit for the annual average concentration of PM_10_ had a short term impact of 91 deaths per year in these cities (AD ranged from 5 per year in Ravenna to 14 per year in Piacenza), corresponding to 5.9 deaths per 100 000 inhabitants per year. Exceeding the WHO limit of 10 μg/m^3^ for the annual average of PM_2.5_, caused 46 deaths per year in the provincial capitals, corresponding to 3.0 deaths per 100 000 inhabitants per year. A negligible short term impact was estimated considering the scenario of EU limit exceedances for daily concentrations of PM_10_. On the contrary, the estimated short term impact when fixing the counterfactual value to the annual average concentration observed in non-urban areas (9.1 μg/m^3^) was very high: 179 AD, corresponding to 11.7 deaths per 100 000 inhabitants per year.

**Table 3:** Estimated number of deaths attributable (AD) to PM_10_ and PM_2.5_ and corresponding attributable community rate (ACR) per 100 000 inhabitants under different counterfactual scenarios by capital city (using the “background” exposure) and total (using different exposure assessment approaches), Emilia-Romagna region (2006–2010)

City^a^	RS1-PM_10_ V = 20 μg/m^3^			RS2-PM_10_ V = 9.1 μg/m^3^			RS3-PM_10_ V = annual average obtained if the EU limit for daily averages is respected			RS1-PM_2.5_ V = 10 μg/m^3^		
	AD	50% CrI	ACR	AD	50% CrI	ACR	AD	50% CrI	ACR	AD	50% CrI	ACR
Piacenza	14	7, 18	14.0	23	11, 29	22.8	2	1, 2	1.7	5	0, 12	4.9
Parma	13	6, 21	7.5	23	10, 36	12.9	1	0, 2	0.6	4	0, 14	2.5
Reggio Emilia	11	6, 16	7.0	21	10, 29	12.6	1	0, 1	0.4	7	0, 16	4.1
Modena	12	4, 22	6.7	20	7, 36	11.1	1	0, 3	0.8	5	0, 15	2.6
Bologna	12	6,19	3.3	35	17, 54	9.5	0	0, 1	0.1	9	0, 22	2.5
Ferrara	7	4, 9	4.9	16	9, 24	12.3	0	0, 0	0.0	6	0, 13	4.2
Ravenna	5	1, 11	3.0	9	1, 22	5.9	0	0, 0	0.0	3	0, 7	1.9
Forlì	6	3, 8	4.9	12	6, 18	10.6	0	0, 0	0.2	2	0, 6	1.8
Rimini	11	6, 16	8.2	20	10, 28	14.1	1	0, 1	0.7	5	0, 13	3.7
9 cities - “Background” exposure^b^	91	41, 140	5.9	179	81, 275	11.7	7	3, 10	0.4	46	0, 119	3.0
9 cities - “Average” exposure^b^	86	19, 150	5.6	141	31, 247	9.2	10	2, 18	0.7	-	-	-
9 cities - “Traffic” exposure^b^	89	21, 155	5.8	143	33, 251	9.4	12	3, 21	0.8	-	-	-

Abbreviations: *V* counterfactual value, *AD* attributable deaths, *CrI* credibility interval, *ACR* attributable community rate;

RS1-PM_10_ = reduction scenario where V is equal to 20 μg/m^3^ annual average (WHO Air Quality Guideline threshold);

RS2-PM_10_ = reduction scenario where V is equal to annual average concentrations observed in non-urban areas;

RS3-PM_10_ = reduction scenario where V is equal to annual average obtained if only 35 days, per year, concentrations exceeding 50 μg/m^3^;

RS1-PM_2.5_ = reduction scenario where V is equal to 10 μg/m^3^ annual average (WHO Air Quality Guideline threshold)

^a^: For each city, “background” exposure and “background “effect estimate were used, with the exception of Ferrara for which the “background” overall meta-analytic estimate was used

^b^: The low/upper limit of the credibility interval was calculated as the sum of the low/upper limits of the city-specific credibility intervals

The results of the sensitivity analysis which compared short term impacts estimated under different exposure specifications are reported in the last three rows of Table 3. We found that AD were largely similar when considering the three different definitions of exposure and the counterfactual value of 20 μg/m^3^: we estimated 86 AD per year (50% CrI: 19, 150) for the “average” exposure, 91 (50% CrI: 41, 140) for the “background” exposure and 89 (50% CrI: 21, 155) for the “traffic” exposure. This was partly expected, because the lowest “traffic” effect estimates were combined with higher air pollution concentrations, whereas the highest “background” effect estimates were combined with lower air pollution concentrations. A certain discrepancy between “background” and “traffic” impacts arose under the counterfactual value of 9.1 μg/m^3^, but the credibility intervals largely overlapped.

The impact estimates at regional level for the year 2010 (using the NINFA-PESCO exposure) are shown in Table 4. The impact estimates in the provincial capitals using the NINFA-PESCO exposure appeared to be similar to those obtained for the period 2006–2010 using data from the monitoring stations. Exceeding the WHO limit of 20 μg/m^3^ for the annual average level of PM_10_ was responsible for 190 deaths per year in the region (AD ranged from 7 per year in cities with less than 5 000 inhabitants to 104 per year in cities with more than 50 000 inhabitants) and exceeding the WHO limit of 10 μg/m^3^ for the annual average level of PM_2.5_ was responsible for 123 deaths per year (AD ranged from 7 per year in cities with less than 5 000 inhabitants to 56 per year in cities with more than 50 000 inhabitants).

**Table 4:** Population weighted (PW)-average concentration of PM_10_ and PM_2.5_ from the NINFA-PESCO model, estimated number of attributable deaths (AD) with 50% credibility interval (CrI), and attributable community rate (ACR) per 100 000 inhabitants under different counterfactual scenarios, Emilia-Romagna region (2010)

	PM_10_ (μg/m^3^)	RS1-PM_10_ V = 20 μg/m^3^			RS2-PM_10_ V = 9.1 μg/m^3^			PM_2.5_ (μg/m^3^)	RS1-PM_2.5_ V = 10 μg/m^3^		
		AD	50% CrI^a^	ACR	AD	50% CrI^a^	ACR		AD	50% CrI^a^	ACR
Emilia-Romagna region	27.3	190	94, 283	4.4	456	229, 675	10.5	18.6	123	0, 304	2.8
nine capital cities	29.8	91	41, 139	5.9	191	86, 292	12.5	19.2	47	0, 123	3.1
Other cities	25.9	99	53, 144	3.5	265	143, 383	9.4	18.3	75	0, 182	2.7
By municipality dimension											
>50 000	29.5	104	48, 158	5.7	222	103, 338	12.2	19.2	56	0, 144	3.1
20 000–50 000	27.5	23	12, 33	4.1	57	31, 82	10.1	19.2	16	0, 39	2.9
10 000–20 000	26.8	31	17, 45	3.7	79	43, 115	9.4	18.9	23	0, 56	2.8
5 000–10 000	26.2	25	13, 36	3.6	66	45, 87	9.4	19.0	20	0, 48	2.8
< 5 000	20.2	7	4, 10	1.7	32	16, 51	7.5	13.7	7	0, 17	1.7

Abbreviations: *V* counterfactual value, *AD* attributable deaths, *CrI* credibility interval, *ACR* attributable community rate;

RS1-PM_10_ = reduction scenario where V is equal to 20 μg/m^3^ annual average (WHO Air Quality Guideline threshold);

RS2-PM_10_ = reduction scenario where V is equal to annual average concentrations observed in non-urban areas;

RS1-PM_2.5_ = reduction scenario where V is equal to 10 μg/m^3^ annual average (WHO Air Quality Guideline threshold)

^a^: The low/upper limit of the credibility interval was calculated as the sum of the low/upper limits of the city-specific credibility intervals

## Discussion

The present work assessed the short-term impact of high concentrations of PM_10_ and PM_2.5_ on mortality in the Emilia-Romagna region. We found that in the cities with more than 100 000 inhabitants, during the period 2006–2010, exceeding the WHO limit for the annual average level of PM_10_ produced 5.9 deaths per 100 000 inhabitants per year. When considering PM_2.5_, exceeding the WHO limit of 10 μg/m^3^ was annually responsible for 3.0 deaths per 100 000 inhabitants.

While the impact results for PM_10_ in the provincial capitals were in line with those in the 25 Italian cities participating in the EpiAir2 project (8.4 deaths per 100 000 inhabitants per year) [24], we found that the ACR due to high PM_10_ levels in the Emilia-Romagna region was lower than that reported in Baccini et al. for the adjoining Lombardy region during the study period 2003–2006 (12.6 deaths per 100 000) [23]. Similarly, the effects and the impacts estimated for PM_2.5_, during the same study period, were lower than the national ones (7.4 deaths per 100 000) [24]. These discrepancies are likely due to the fact that our meta-analysis and the cited ones refer to cities that are heterogeneous both in terms of socio-demographic characteristics and exposure levels, use different statistical approaches and focus on different calendar time periods [11].

Our analyses highlighted also an important impact in medium and small-sized municipalities in the region. However, in interpreting this result, it has to pointed out that we extended the overall effect estimate of the meta-analysis on the capital cities to the whole region, including smaller and less polluted areas, so that the presence of a certain degree of bias cannot be ruled out. For PM_10_, 48% of AD were estimated among people residing in the nine capital cities, where more than 35% of the regional total population lives. For PM_2.5_, this percentage decreased at 38%, because of the lower heterogeneity of the PM_2.5_ concentrations among large and small municipalities. ACRs for small and medium-sized municipalities (between 5 000 and 50 000 inhabitants) were lower than ACRs for cities with more than 50 000 inhabitants, but not negligible, indicating that the impact was also relevant in the smaller municipalities.

Selecting the “appropriate” monitors is part of the more general issue concerning the use of fixed-site stations to measure population exposure. This issue is related to the problem of exposure misclassification and its effects on the estimated associations [44], but discussing these aspects was beyond the aim of this paper. Objective of the present study was to evaluate the consequence of selecting monitors according to their classification (as background or traffic stations) on effect and impact estimates.

It should be noticed that in general the usual classification of monitors in traffic and background stations could be not informative regarding their ability to measure the actual exposure of the resident population. Usually the air pollutants levels measured by background monitoring stations are considered more appropriate to represent population exposure, but this could not be the case if these monitors are located in urban parks, far from residence areas. On the other hand, traffic monitoring stations could be accounted for if located close to residential areas, even if they generally reflect hot spots of urban pollution.

We found that the PM_10_ effect estimates were not robust to different exposure assessment approaches. The estimated percentage variation on the capital cities was higher when considering the exposure from background monitors than when considering the exposure from traffic monitors. This result was partly expected due to the nature of the regression model and the data pattern. In fact, the correlations between traffic and background daily concentrations were very high (0.92 on average), indicating that both time series were capturing the same underlying phenomenon. This high correlation, coupled with the fact that the ratio between background values and traffic values was always lower than 1 (with the exception of Ravenna), was consistent with the observed ratio between the percentage variations estimated by the Poisson regression models when the two different exposures were used.

A second possible explanation of the observed discrepancy between effect estimates is that higher daily pollutant levels (such as those usually measured by traffic sites) could be actually associated with lower effects, indicating non-linearity of the exposure-response curve on a log scale. However, it seems difficult to conclude for non-linearity from analyses conducted on general population without focusing on subgroups of people actually exposed to the air pollutant levels measured by the different monitoring stations.

Finally, we cannot exclude that the observed discrepancy between traffic and background percentage variations was partly due to a larger degree of exposure misclassification when using traffic monitors than when using background monitors to assess the exposure level of the resident population. This larger misclassification could have brought to a certain degree of underestimation of the estimated associations.

Roemer and van Wijnen found results in line with ours, reporting larger effects using background stations rather than the traffic ones, when considering black smoke, CO, NO, NO_2_ and SO_2_ [45]. Also these authors conclude that the lower relative risks associated to traffic concentrations is likely due to the larger range of variation of these measures in respect to the background ones and to exposure misclassification.

In our study we focused also on the sensitivity of the impact estimates to different exposure assessment. Despite the discrepancy in terms of percentage variations, when exposure levels and effect estimates were combined to calculate AD, in particular when the counterfactual value of 20 μg/m^3^ for PM_10_ was considered, we found that the impact estimates essentially overlapped. In fact, the lower percentage variations estimated for the traffic monitors were coupled with the higher levels of exposure measured by these monitors, and the opposite happened for the background monitors. This indicates a substantial robustness of our impact estimates to different exposure definitions. This robustness also strengthens our results at regional level, when we used the “background” exposure, the only one assuring a homogeneous definition of the air pollutant levels over the region.

It should be stressed that the substantial robustness of the impact estimates relied on the fact that we used the same exposure measures both in effect estimation and impact calculation. This consistency could decay in those studies where impact is assessed by combining effect estimates from the literature with actual air pollutant levels. In our study, combining effect estimates with incongruous exposures would have brought to substantial differences in terms of ACR. For example, when considering the WHO reduction scenario for PM_10_ (RS1-PM_10_), the overall ACR in the nine capital cities would have been equal to 9.4 if “background” effect estimates and “traffic” exposures had been used, and equal to 3.7 if “traffic” effect estimates had been combined with “background” exposures. On the contrary, using the same exposures measure both in effect and impact estimates provided very similar impacts: ACRs were equal to 5.9 and 5.8 under the “background” approach and the “traffic” approach, respectively (Table 3).

## Conclusion

In conclusion, our study confirmed that a reduction in particle concentrations during the study period would have produced significant health benefits in all municipalities of the Emilia Romagna region, from the largest cities to the smallest towns. While the effect estimates were sensitive to the use of different exposure assessment approaches, the impact estimates were more robust, provided that the same exposure definition was used both for effect estimation and attributable deaths calculation. This highlights that caution is required in using effect estimates obtained from the literature to estimate actual impacts, without considering how exposures were calculated.

## Abbreviations

ACR: Attributable community rate
AD: Attributable deaths
ARPAE-ER: Environmental Protection Agency of Emilia-Romagna region
CrI: Credibility interval
EU: European Union
ICD: International classification of disease
PM_10_: Particulate matter ≤10 μm in diameter
PM_2.5_: Particulate matter ≤2.5 μm in diameter
PW: Population-weighted
RS: Reduction scenario
WHO: World Health Organization
